# Penetrating extremity injuries in a large Scandinavian Level-1 trauma center: a 4-year analysis of imaging methods, mechanisms of injury and clinical outcome

**DOI:** 10.1007/s00590-025-04507-x

**Published:** 2025-09-10

**Authors:** Paulina Cewe, Sigurveig Thorisdottir, Gudrun L. Oladottir, Victor Gabriel El-Hajj, Victor E. Staartjes, Adrian Elmi-Terander, Seppo K. Koskinen, Erik Edström

**Affiliations:** 1 Department of Clinical Neuroscience, Karolinska Institute, Stockholm, Sweden; 2 Department of Trauma and Musculoskeletal Radiology,Karolinska University Hospital, Stockholm, Sweden; 3 Capio Spine Center Stockholm, Löwenströmska Hospital, Upplands Väsby, Sweden; 4 Machine Intelligence in Clinical Neuroscience & Microsurgical Neuroanatomy (MICN) Laboratory, Department of Neurosurgery, Clinical Neuroscience Center, University Hospital Zurich, University of Zurich, Zurich, Switzerland; 5 Department of Surgical Sciences, Uppsala University, Uppsala, Sweden; 6 Department of Medical Sciences, Örebro University, Örebro, Sweden

**Keywords:** Trauma, Injuries, Penetrating, Extremities, Vascular, CT, Angiography, Imaging

## Abstract

**Background:**

To analyze penetrating extremity injuries at a Scandinavian urban Level-1 trauma center regarding incidence, mechanism of injury, imaging approach and clinical outcome.

**Methods:**

A retrospective study (2013–2016) of penetrating injuries to the extremities based on a Trauma Registry. Retrieved variables included patient demographics, injury characteristics, time to CT and 30-day morbidity.

**Results:**

Of 636 patients with penetrating trauma, 142 (22.3%) sustained injuries to the extremities that required imaging, surgical treatment, or observation. Median age was 25 years (15–83) and consisted mostly of males (90.8%, 129/142). Most were Level-1 trauma, 81.7%, with 28.9% multi trauma. Most common injuries were gunshot wounds (GSW, 47.9%) and stab wounds (SW, 47.2%), and lower extremities were most common (77.8%). Imaging showing arterial injury was present in 31.0%. %. CT-angiography was the most common modality 65.5% (93/142) and had excellent sensitivity for vascular injury of 100%. More than half of CTAs were negative: 60.4% in GSW compared to 53.7% in SW. GSWs were 7.4-times likelier to necessitate acute surgery compared to SWs (*p* = 0.0001). Surgery requiring vascular repair consisted of 19.0% and was more common in GSW 11.3% compared to SW 6.3%. Fasciotomy was performed in 5.6% of all GSW. In 1.4% there was loss of limb. The 30-day morbidity rate was 16.2%, mortality was 0%. Overall, GSW increased the odds for 30-day complications, compared to SW (Odds-ratio = 14.1, *p* < 0.001).

**Conclusions:**

In a Scandinavian Level-1 Trauma Center, about every fourth to fifth admission for penetrating trauma involved penetrating extremity injuries. GSWs had a significantly higher probability for acute surgery, and higher rate of 30-day complications. The rate of arterial injury was around one third of all injuries. CTA is effective and sensitive in detecting clinically relevant vascular injuries. Half of CTAs were negative. Stratifying patients into exam-based indication for CTA is important to reduce unnecessary imaging.

## Introduction

Worldwide, traumatic injuries are a leading cause of death and disability for individuals under the age of 45 [1]. The prevalence of penetrating trauma, notably gunshot wounds (GSW), in Sweden has risen significantly compared to other similar European nations. Penetrating trauma to the extremities is of concern due to the potential for significant blood loss, severe damage, and long-term debilitating consequences. These types of injuries can occur from various causes, including projectiles (e.g., bullets from guns, blast fragments) or stabs (e.g. knifes, scissors, glass objects), and can cause fractures, nerve damage and potentially life-threatening vascular injuries [2, 3]. Particularly, the assessment of arterial damage is crucial for early intervention and optimal patient outcomes [2, 3].

Depending on the hospital facility, various imaging modalities can be utilized in the trauma setting for diagnosing traumatic injuries, such as Computed Tomography (CT), Digital Subtraction Angiography (DSA), conventional X-ray (XR) and Doppler Ultrasound (US). Invasive, catheter-based imaging (DSA) has been the gold-standard for diagnosing vascular injuries to the extremities, however, has been substituted by CT angiography (CTA) in recent years as primary imaging [4, 5]. CTA of the extremities is either performed alone, or as an added protocol to contrast-enhanced whole-body computed tomography (WBCT). It acquires information fast and reliably regarding injury severity and potential arterial damage [6]. Furthermore, the use of CTA and WBCT saves time spent in the emergency department, facilitates faster time to diagnosis and triage so that proper treatment is not delayed [6–8]. Indication of CTA should be in accordance with relevant findings on the physical exam. However, with increasing availability there has been a steady increase in CT scans, with overutilization and unnecessary imaging [9, 10]. The potential dangers associated with ionizing radiation should be taken into consideration and adhering to the ALARA (as low as reasonably achievable) principle, should be considered [4, 8, 10].

The radiological signs on CTA of arterial vessel injury are active extravasation of contrast media (CM), or abrupt loss of CM in occlusion, abnormal vessel filling of veins in arterio-venous (AV) fistulas, focal bulging in pseudoaneurysms, transections with extravasation of CM, and various contour abnormalities such as in dissections or vessel spasm, of which the latter is common in younger patients and does not have to be a sign of vessel injury [4, 5, 11]. Regarding optimal visualization of subtle vascular injuries, a minimum CM enhancement of usually 150 Hounsfield Units (HU) is needed [12]. Furthermore, caution should be taken when foreign objects with high HU values, such as metal and bullet fragments remain in the injured area, as well as with adjacent orthopedic prosthetics, since these objects can produce streaking artifacts such as photon starvation and beam-hardening which limit evaluation of potentially injured vessels [11–13].

Immediate surgical intervention is warranted when imaging reveals critical findings in major arteries, such as the presence of contrast extravasation, early pseudoaneurysm formation, or occlusion. However, if extravasation, AV fistula, or occlusion occurs in an artery not classified as one of the major, a conservative approach involving observation or therapeutic embolization (for AV fistula or extravasation) may be considered. When imaging identifies an intimal defect, it is important to note that invasive vascular intervention is typically unnecessary, as a substantial percentage of such defects tend to heal spontaneously without the need for surgical intervention. In contour abnormalities suggestive of peripheral artery spasm without findings of ischemia, which is more common in younger patients, a conservative approach with observation is warranted. It is essential to remain vigilant for potential signs that may require ongoing assessment and monitoring, including compartment syndrome, in situ thrombosis, or distal embolism [4, 14].

We aim to analyze the incidence of penetrating extremity injuries during the study period in a large Scandinavian Level-1 trauma center, and to evaluate the imaging approach, image quality, mechanisms of injury, injury locations, age/sex demographics, and 30-day morbidity and mortality.

## Material and methods

### Overview and inclusion criteria

All patients with penetrating trauma during a four-year period from 1 January 2013 to 31 December 2016, were identified and retrieved from the local trauma registry database (TRD) of a major level 1 Scandinavian trauma center, with a catchment area of 3 million inhabitants.

Patients who met the following criteria were eligible for enrollment in the study: Age 16 years or older, traumatic injury caused by penetration to upper and lower extremities. All traumatic injuries, all body regions, superficial and deep, were included. Exclusion criteria was no corresponding imaging in the local PACS or misclassified injury. Data of patients that died before hospital arrival was not accessible.

### Data collection

Parameters that were extracted from the TRD were: Age, gender, Injury Severity Score (ISS), New Injury Severity Score (NISS), time to first CT, length of hospital stay, and 30-day mortality. All radiological images, and HU values were retrieved from local picture archiving and communication systems (PACS; SECTRA v. 19.3.11, SECTRA AB, Linkoping, Sweden).

Imaging was performed with two commercially available devices: The Revolution multidetector CT (GE Healthcare, Milwaukee, WI, USA) a 256-detector row scanner, and the LightSpeed VCT GE Healthcare, Milwaukee, WI, USA) a 64-row scanner. Protocols used were WBCT with added extension of CTA of the extremities, standalone CTA, contrast enhanced CT in venous phase (CECT) and non-contrast enhanced CT (NECT). In both WBCT and CTA an additional venous phase could be added. Tube current and voltage was 150 mA and 100 kV in the arterial phase and 120 mA and 120 kV in the venous phase respectively. A higher tube current and voltage was used for large patients, 300 mA and 100 kV and 255 mA and 120 kV in the arterial and venous phase respectively.

Depending on site of injury, the region of interest (ROI) was set either in the thoracic or abdominal aorta respectively, with 150 HU as the scan timing value. The timing of contrast enhancement was automatic in all imaging exams, using SmartPrep software (GE Healthcare, Milwaukee, WI, USA). In WBCT and CTA, when both arterial and venous IV protocols were initiated, delay was set to 45 s starting from the end of the arterial phase. Rotation time was 0.5s and 0.4s for Revolution CT and LightSpeed VCT respectively. All protocols independent of detector model were conducted with a pitch of 0.992:1.

Two radiologists reviewed the TRD for initial collection of patients for the following: If corresponding imaging in PACS was available, and the localization of injured body parts. For patients with penetrating injury localized to the upper and lower extremities, further assessment was made by one trauma radiologist, with the support of a senior trauma radiologist and a senior interventional radiologist, findings were compared to the initial reports. Classification of injury type into 3 groups was made, GSW, SW and other injuries. The injury site for upper extremities was located between the mid-clavicle and the hand and for lower extremities between the inguinal ligament and the foot. For each patient the MPR-view was used for review, as well as 2D- and 3D-reformations. Assessment was made of imaging modality, presence of metal foreign bodies such as metal- or bullet fragments on CT scout images and XR-images, and presence of vessel injuries and fractures. Furthermore, to assess sufficient image quality, the HU value was obtained by measuring the region of interest (ROI) in two anatomical vessel locations at the level of injury. For lower extremity exams, the proximal ROI was placed in the femoral artery at the inguinal level and a second distal ROI placed 10 cm proximally of the injured vessel site. For the upper extremity exams, the proximal ROI was placed in the axillary artery at the mid-axillary line and a second distal ROI placed 10 cm proximally of the injured vessel site. Attenuation measurements below 150 HU were considered diagnostically limited. Artifacts were recorded, and if a vessel was either partially or totally obscured, the case was classified as diagnostically limited.

Vessel injuries were categorized according to radiological findings: Active arterial extravasation, occlusion, transection, AV-fistula, pseudoaneurysm, and dissection. Active arterial extravasation was defined as extravascular CM with HU values similar to, or higher than that of the nearest largest artery such as the femoral or axillary depending on injury location. Occlusion was defined as abrupt termination of an artery, without patent lumen distally. Transection was defined as abrupt termination of an artery with extravascular CM. AV-fistula was defined as early filling of a venous vessel, regardless of whether the fistulous connection was observed. Pseudoaneurysm was defined as an irregular vessel protrusion of an artery, with smooth margins, that communicated with the vessel lumen. Dissection was defined as irregular caliber of an arterial segment with preserved luminal patency, compared both proximal and distal to the irregular segment, with either wall thickening or thrombus. Subtle contour abnormality was classified as vessel spasm and not included as vessel injury.

Patient medical records were recorded to acquire information such as primary survey findings in the trauma bay, use of ABI-index, treatment strategies, surgical operation method, and 30-day morbidity. Surgery was classified as endovascular, exploratory with vascular repair, or exploratory without vascular repair. Conservative treatment was classified as superficial surgery with wound sutures. Outcome was documented based on the subsequent clinical management and outcome within the 30 days following the initial imaging. Assessment was made regarding imaging modalities, image quality, injury characteristics, treatment strategies and outcomes between GSW and SW.

This study was approved by the Institutional Review Board which also waived the need for informed consent. There are no conflicts of interest.

### Statistical analysis

Data preparation and statistical analysis was done with software package R-Studio v. 2023.09.0 + 463. Incidence of injury localization and types, imaging methods, positive findings for vessel injury, and need for surgical management was analyzed between GSW and SW using a chi-square test. Odds Ratio (OR) and 95% Confidence Interval (CI) were calculated. *P*-values ≤ 0.05 were considered statistically significant. Descriptive statistics, including mean, median, and percentage were used to describe numerical data.

## Results

### Patient demographics

**Fig. 1:**
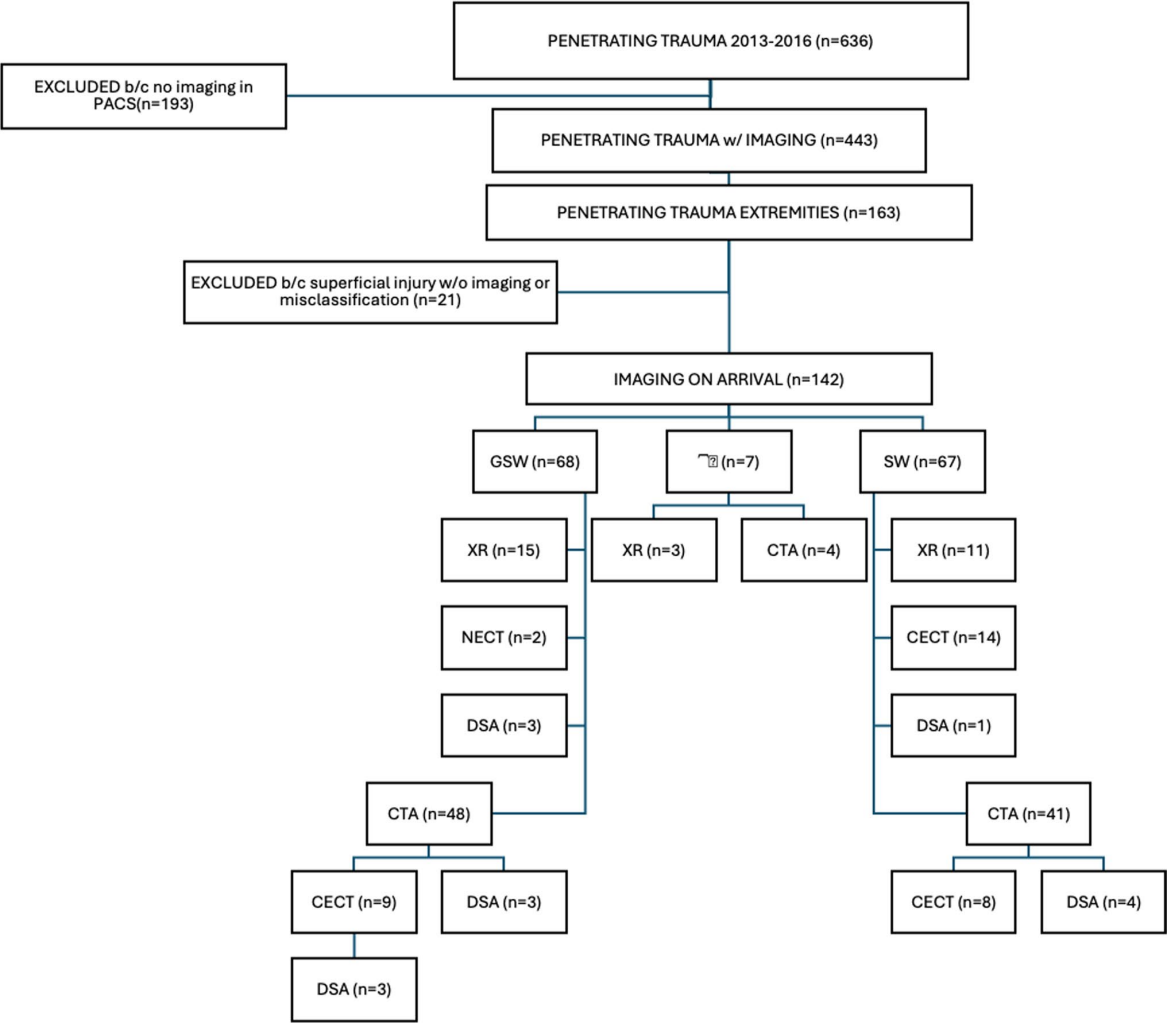
Cohort identification with exclusion criteria of patients included in the study, and imaging flowchart of modalities in penetrating extremity trauma: CTA, CT Angiography; CECT, Contrast enhanced CT in venous phase; DSA, Digital Subtraction Angiography; NECT, non-enhanced CT; GSW, Gunshot wound; SW, Stab wound; XR, conventional X-ray imaging

During the four-year study period, there were 636 cases of penetrating trauma reported in the TRD. From this group, 443 cases had imaging studies in the local PACS of which a subgroup of 142 (22.3%) patients met the inclusion criteria for this study (Fig. 1). These patients sustained penetrating extremity injuries and underwent imaging using one or more modalities. Most of the patients were male (129 men, 90.8% and 13 women, 9.2%). The median age was 25 years (15–83 years). Most cases resulted from interpersonal violence, of which 68 (47.9%) were gunshot wounds (GSW), 67 (47.2%) from stab wounds (SW), and 7 (4.9%) attributed to miscellaneous penetrating injuries classified as “other wounds”. Self-inflicted trauma was found in 2.2% (3/142), comprised of GSW 0.7% (1/142) and SW 1.4% (2/142), comprised of all men (Table 1).

**Table 1:** Demographic and clinical characteristics of penetrating extremity injuries. Values are given as n (%) or median (range)

Demographics	Overall	Gunshot	Stab	Other
Number of patients (n (%))	142 (100)	68 (47.9)	67 (47.2)	7 (4.9)
Age median (median (range))	25 (15–83)			
Male (n (%, median age, age range))	129 (90.8, 25, 15–83)	61 (43.0)	62 (43.7)	6 (4.2)
Female (n (%, median age, age range))	13 (9.2, 24, 18–46)	6 (4.2)	6 (4.2)	1 (0.7)
*Injury characteristics*				
Lower extremity injury (n (%))	110 (77.8)	63 (44.4)	42 (29.6)	5 (3.5)
Upper extremity injury (n (%))	48 (33.8)	14 (9.9)	30 (21.1)	4 (2.8)
Multi-trauma injuries	41 (28.9)	15 (10.6)	23 (16.2)	3 (2.1)
Self-inflicted trauma (n (%))	3 (2.2)	1 (0.7)	2 (1.4)	
Self-inflicted trauma (M:F)	3:0			
ISS median (median (range))	5 (–38)	9 (–29)	2 (1–35)	2 (1–38)
NISS median (median (range))	6 (1–43)	9 (1–34)	3 (1–43)	3 (1–43)
Days in hospital (median (range))	3 (1–62)	3 (1–49)	2 (1–33)	6 (2–62)
*Trauma level activation*				
Level 1 (n (%))	116 (81.7)	62 (43.7)	50 (35.2)	4 (2.8)
Level 2 (n (%))	26 (18.3)	6 (4.2)	17 (12.0)	3 (2.1)
Time to CT (median (range))	29:30 (00:15:00–08:12:00)			
*Treatment strategy and outcome*				
Hard signs for vessel injury (n (%))	19 (13.4)	9 (6.3)	7 (5.0)	3 (2.1)
Osseus injuries	35 (24.6)	30 (21.1)	2 (1.4)	3 (2.1)
Acute surgery (n (%))	61 (43.0)	44 (31.0)	13 (9.2)	4 (2.8)
Surgery with vascular repair (n (%))	27 (19.0)	16 (11.3)	9 (6.3)	2 (1.4)
30-day morbidity (n (%))	21 (14.8)	16 (11.3)	3 (2.1)	2 (1.4)
30-day mortality (n (%))	0			

*ISS* Injury severity scale, *NISS* New injury severity scale, *CT* Computer tomography

Imaging of the lower extremities was most common with 77.5% (110/142) cases compared to 33.8% (48/142) of the upper. Isolated injuries to the lower extremities amounted to 57.0% (81/142) and the upper 14.1% (20/142). Multi trauma injuries in total amounted to 28.9% (41/142), of which the abdomen was the most common combined anatomical region in 12.7% (18/142). Other anatomical regions in multi trauma injuries were thorax 10.0% (14/142), neck 4.2% (6/142), head 2.1% (3/142) and spine 1.4% (2/142). In 21.1% (30/142) cases of multi trauma both upper and lower extremities were injured.

Most of the cases were documented as Level-1 trauma, 81.7% (116/142). Level-2 trauma comprised 18.3% (26/142) of cases. The median overall NISS for the GSW group was 9 (1–34), compared with median NISS 3 (1–43) for the SW group. Median NISS for cases with positive imaging findings of vascular injuries was 12 (2–34) for GSW and 10 (1–29) for SW, suggesting a higher severity of GSW injuries compared to SW.

### Vascular injuries

Of the total cohort of 142 patients with penetrating injuries to the extremities, 44 (31.0%) had positive findings of vascular injury (Table 2), which consisted of 15.5% (22/142) GSW, 14.1% (20/142) SW, and 1.4% (2/142) other injuries. Most prevalent finding was active arterial extravasation 63.6% (28/44) either alone or in combination with, transection 13.6% (6/44), occlusion 9.1% (4/44), dissection 9.1% (4/44), pseudoaneurysm 6.8% (3/44), and AV-fistula 6.8 (3/44). Forty (43.0%) of 93 patients imaged with CTA were positive for vascular injury on CTA, and comprised of 20.4% (19/93) GSW, 20.4% (19/93) SW, and 2.2% (2/93) other injuries.

**Table 2:** Distribution, localization of arterial injuries, injury types, and complications within 30 days

	Overall	Gunshot	Stab	Other injuries
*Artery*				
Superficial femoral	10	8	2	
Profunda femoris	10	4	6	
Profunda femoris, branch	5	1	4	
Popliteal	3	2		1
Popliteal, branch	5	3	2	
Anterior tibial	3	3		
Posterior tibial	3	1	2	
Peroneal	2	2		
Axillary, branch	2		2	
Brachial	2		2	
Ulnar, branch	2	2		
Palmar digital	1			1
*Injury type*				
Extravasation	28	12	15	1
Transection	6	3	2	1
Occlusion	4	2	2	
Dissection	4	4		
AV-fistula	3			
Pseudo-aneurysm	3	1	2	
*Complications*				
Wound revision	6	5		1
Infection	4	3	1	
Nerve damage	7	5	2	
Amputation	2			2
AV-fistula	2	1	1	
Pseudo-aneurysm	1	1		
Fasciotomy	8	7		1

*AV* Arterio-venous

### Osseus injuries

Of the 142 patients with penetrating trauma, osseous injuries were present in 24.6% (35/142) cases, and more common in GSW 21.1% (30/142) vs. SW 1.4% (2/142), and 2.1% (3/142) other injuries respectively.

### Bullet fragments

Of the total 109 CT scout scans performed, including CTA, CECT and NECT, bullet fragments were found on 29.4% (32/109) scouts. One patient imaged with only XR of the lower extremity had bullet fragments on scan. Of the total 33 patients with bullet fragments on imaging, almost all 90.9% (30/33) underwent acute surgery.

### CT imaging protocols

The average amount of administered intravenous CM was slightly higher in WBCT with CTA, 127.1 mL (range = 90–200 mL, median 125 mL) compared to CTA of extremities only, 116.5 mL (range = 85–120, median 120) and with CECT 110.8 mL (range = 70–200 mL, median 100 mL). The IV contrast injection speed was 4.5–6.0 mL/s and 2.5–3.5 mL/s in arterial and venous phase respectively. Wounds were marked with vitamin-E capsules prior to CT imaging to simplify subsequent radiological evaluation, of which half of the cases (71/142) were marked. Time to imaging, from arrival to hospital to initial CT was assessed as marked time on CT scout image and corresponded to median of 29.30 min (range 00:15:00–08:12:00).

### Image quality

Image quality was evaluated by placing two ROIs, one in the nearest largest vessel and one proximally of the injured vessel site and measuring HU. Vessel opacification in CTA was sufficient in all exams, with a median HU of 356 (range 150–616). Diagnostic limitations were found in 4 CTAs because of partial Tourniquets during the CT scan, both were GSW to the lower extremities. Subsequently, in 3 cases abnormal fillings of venous structures were observed but did not fulfill radiological classification as AV-fistulas but likely related to clinical data of previous use of Tourniquets. Bullet and skeletal fragments limited soft-tissue assessment and discrete vessel injuries due to streaking artifacts in 3 patients, however none interfered with assessment of substantial vessel injuries.

### Imaging approach and diagnostic capability

**Fig. 2:**
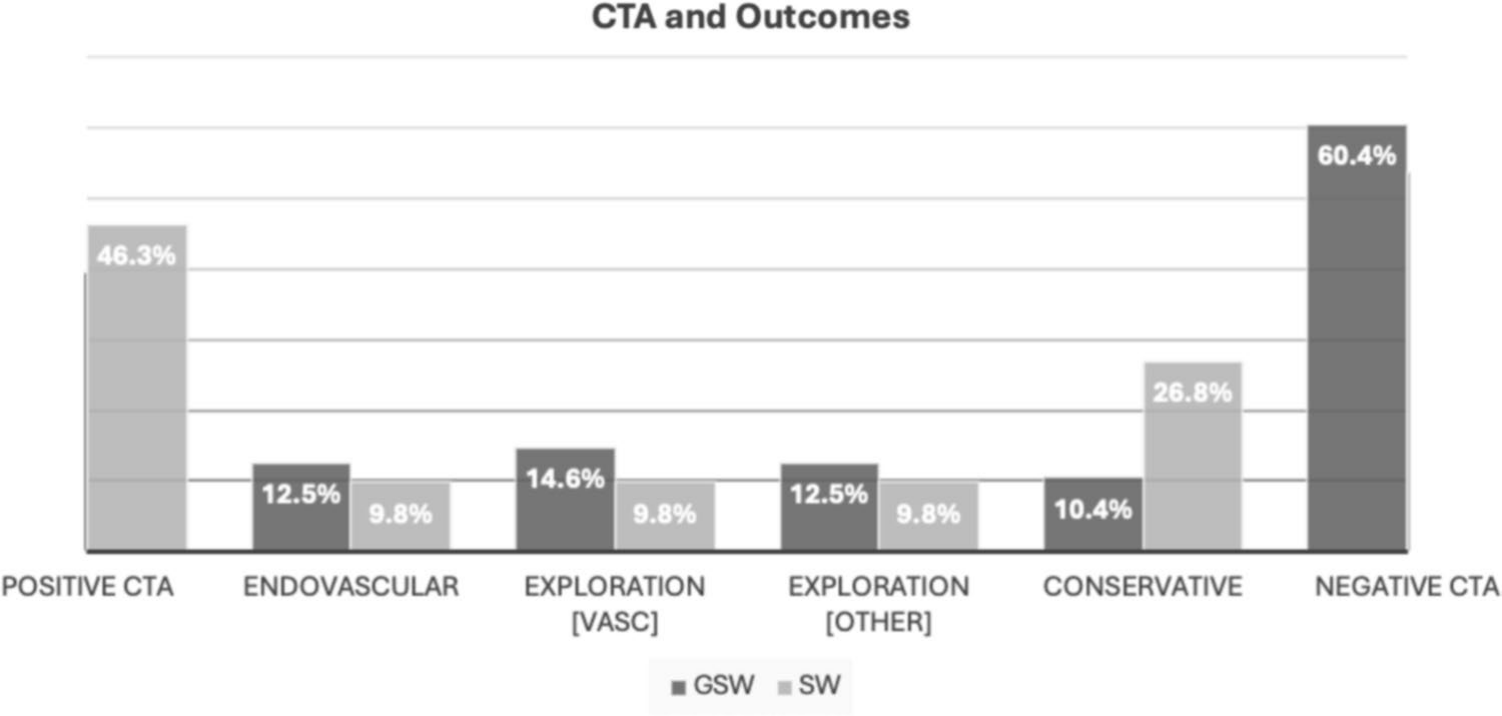
Diagram with frequency of positive CTA in % for vascular injury, frequency of surgery, conservative treatment, and negative CTA in penetrating extremity trauma. Surgery includes ENDOVASCULAR, endovascular repair with graft or coiling; EXPLORATION [VASC], exploratory surgery with vascular ligation or diathermy; EXPLORATION [OTHER], exploratory surgery without vascular repair; GSW, Gunshot wound; SW, Stab wound

Of the 142 patients with penetrating trauma to the extremities, the most prevalent modality utilized was CTA, 65.5% (93/142). The injury mechanism imaged with CTA was GSW in 51.6% (48/93), SW in 44.1% (41/93) and 4.3% (4/93) in other injuries. In 21.5% (20/93) a CT with contrast in venous phase (CECT) was added. Anatomical region most evaluated with CTA was the lower extremities, which comprised of 90.3% (84/93) compared to 22.6% (21/93) of the upper extremities. Of the 93 CTAs performed, 57.0% (53/93) were negative for vascular injury (Fig. 2). This amounted to 60.4% (29/48) GSW, 53.7% (22/41) SW, 50% (2/4) other injuries. None of these cases required acute surgery or vascular intervention and were all managed through conservative treatment.

Subsequently, the data collected from the patient reports, surgical findings, and CTA assessment of all penetrating injuries to the extremities, resulted in 23 true-positive, 19 false-positive, 51 true-negative, and 0 false-negative cases. Overall sensitivity (n = 93) was 100.0% (95% confidence interval CI  =  85.69–100), specificity 72.86% (95% confidence interval CI  = 8 61.46–81.88). The overall positive predictive value (PPV) was 54.76%, and negative predictive value (NPV) of 100.0%.

Sensitivity was 100% for both GSW and SW (Table 3). GSW had higher specificity compared to SW (82.86% vs. 66.67%). The positive predictive value (PPV) was higher in GSW compared to SW, 68.48% vs. 42.11% respectively. Negative predictive value was 100% in GSW and SW.

**Table 3:** Diagnostic performance of CT-Angiography in detecting arterial injury. Values with 95% confidence intervals are provided

	Sensitivity (%)	Specificity (%)	PPV (%)	NPV (%)
Overall	100 (85.69–100)	70 (58.46–79.46)	52.27(37.94–66.25)	100 (92.73–100)
Gunshot	100 (77.19–100)	71.43 (54.95–89.67)	56.52 (36.81–74.37)	100 (86.68–100)
Stab	100 (67.56–100)	66.67 (49.61–80.25)	42.11 (23.14–63.72)	100 (74.12–100)

*NPV* Negative predictive value, *PPV* Positive predictive value

CECT alone was performed in 9.9% (14/142) cases. All cases comprised of SW as injury mechanism. Anatomical region most evaluated with CECT was the upper extremities, which comprised of 8.5% (12/142) compared to 1.4% (2/142) of the lower extremities.

Non-enhanced contrast CT (NECT) was performed in 1.4% (2/142) cases. All of these comprised GSW as injury mechanism, and all constituted of lower extremities. Conventional X-ray (XR) was performed in 20.4% (29/142) cases, comprised of 10.6% (15/142) GSW, 7.7% (11/139) SW, and 2.1% (3/142) other injuries. Most evaluated anatomical region with XR was the lower extremities with 51.7% (15/29) compared to 37.9% (11/29) upper extremities. DSA was performed as primary imaging in 2.8% (4/142) patients, 2.1% (3/142) GSW and 0.7% (1/142) SW, all lower extremities.

### Treatment strategies

**Fig. 3:**
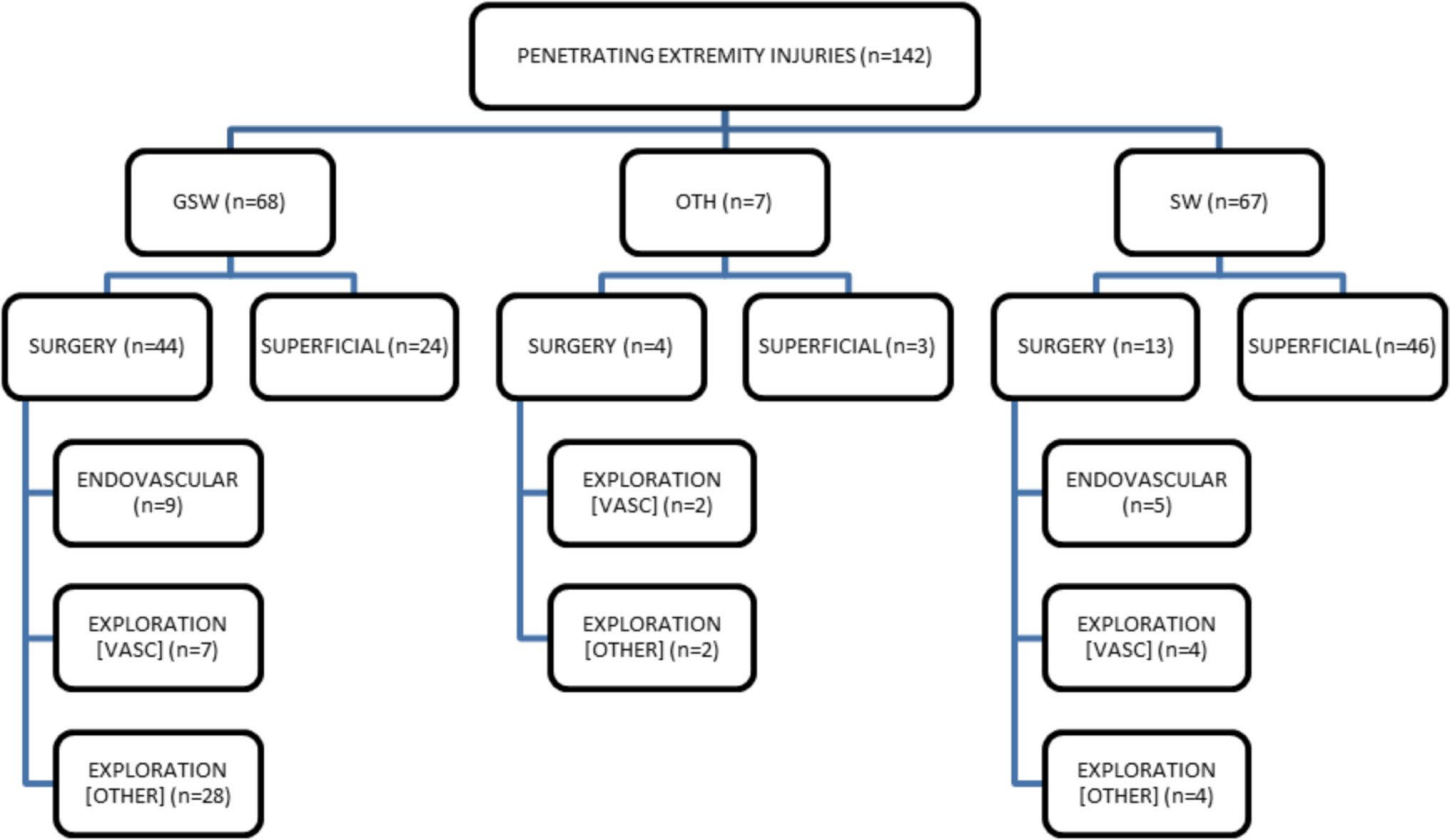
Flowchart of treatment strategy in penetrating extremity trauma for total patient cohort. Surgery includes ENDOVASCULAR, endovascular repair with graft or coiling; EXPLORATION [VASC], exploratory surgery with vascular ligation or diathermy; EXPLORATION [OTHER], exploratory surgery without vascular repair; Superficial, wound closure only. Injuries; GSW, Gunshot wound; SW, Stab wound; OTH, Other injuries

Overall, acute surgery including endovascular intervention, exploratory surgery with and without vascular repair was performed in 43.0% (61/142) of all penetrating extremity trauma, and comprised of 31.0% (44/142) GSW, 9.2% (13/142) SW, and 2.9% (4/142) other injuries (Fig. 3). Most of the exploratory surgeries comprised of wound debridement, extraction of bullet fragments, and removal and repositioning of osseous injuries, and was most common in cases of GSW, accounting for 19.7% (28/142), followed by SW, at 2.8% (4/142), and other injuries at 1.4% (2/142). Superficial injuries, that were treated conservatively with wound closure, were more common in SW compared to GSW, 38.0% (54/142) vs. 19.0% (27/142). Overall, GSW increased the odds for acute surgery by a factor of 7.4 (*p* = 0.0001) compared to SW.

Hard signs of vessel injury were recorded in 13.4% (19/142) cases, 6.3% (9/142) GSW, 5.0% (7/142) SW, 2.1% (3/142) other injuries. All were treated with acute surgery, of which 89.5% (17/19) required vascular repair. Information on ABI Index was found in 21.8% (31/142) patient records. Of these 35.5% (11/31) had abnormal findings, all required acute surgery, of which 25.8% (8/31) required vascular surgery. Of the total osseus injuries, 11.4% required vascular repair, consisting of 5.7% (2/35) GSW and 5.7% (2/35) other injuries.

Complications within 30 days comprised of combinations of recurrent wound revision 4.2% (6/142), persistent nerve damage 4.9% (7/142), infections 2.8% (4/142), AV-fistula 1.4% (2/142), pseudoaneurysm 0.7% (1/142), and traumatic amputation 1.4% (2/142). Fasciotomy because of compartment syndrome was performed in 5.6% (8/142) cases, 7 had prior positive CTAs for vascular injury, and one was imaged with only XR with cortical fracture and bullet on scan. Compartment syndrome developed in 2.8% (4/142) after endovascular treatment, and 2.1% (3/142) after exploratory surgery without vascular repair, all cases consisted of GSW.

Overall mortality rate was 0% (0/142). Complications after positive findings for vessel injury on CTA were more prevalent in GSW compared to SW, 10.6% (15/142) vs. 2.8% (4/142). GSW increased the odds for complications compared to SW by a factor of 14.1 (*p* ≤ 0.001).

## Discussion

While rarely a cause of death, penetrating injuries to the extremities may require rapid surgical intervention to lower the risk of severe morbidity and loss of function [9]. The demographic is often a younger and previously healthy population and there is a considerable societal cost related to hospital stay, rehabilitation, and on the individual level can render substantial loss of function and number of working years [10]. The purpose of this study was to examine the differences in imaging, treatment strategies, and outcomes in penetrating injuries to the extremities in major Level-1 trauma center.

In the trauma setting WBCT and CTA at our institute is ordered in conjunction to the primary survey in the trauma bay, or in the emergency room, based on relevant physical exam indication. Decision on ordering a CTA is at the attending or resident surgeon’s or emergency physician’s discretion, with a set of specific criteria based on a physical exam, to guide in appropriate management of injuries according to the proposed protocols of the Western Trauma Association [15].

Performing CTA either alone, or in conjunction to WBCT when assessing penetrating vascular injuries, assists in faster time to diagnosis and saves time for the patient spent in the emergency department, and overall hospital stay, all in accordance to European guidelines [13]. In our study, median time to CT was 29.30 (00:15:00–08:12:00) minutes, and median hospital stay 3 days (1–62). Included in the median time to CT were patients that were taken to the OR directly after primary survey, with subsequent added OR-time, which may reflect in the higher median time.

Most cases were documented as Level-1 trauma 81.7%, with full trauma team activation consisting of trauma surgeon, orthopedic surgeon, anesthesiologist, and radiologist. A slightly higher severity of GSW was found overall. Acute overall surgery including both vascular repair and exploratory surgery, was performed in 43.0% of all penetrating extremity trauma, and was more common in GSW compared to SW, 31.0% vs. 9.2%. Superficial injuries, that were treated conservatively with wound closure, were more common in SW compared to GSW, 38.0% vs. 19.0%. Overall, GSW increased the odds for acute surgery by a factor of 7.4 (*p* = 0.0001) compared to SW.

The most often proposed imaging was CTA and was utilized in 65.5% of all patients admitted for penetrating trauma to the extremities. Positive CTA for vascular damage was found in 43.0% of CTAs, of which approximately half had arterial damage confirmed during surgery, consisting of 68.4% GSW, 21.1% SW cases and 10.5% other injuries. Vessel opacification in CTA was sufficient in all exams, with a median HU of 356 (range 150–616). Diagnostic limitations were found on 7 CTAs, all GSW, 4 because of partial Tourniquets and 3 because of streaking artifacts from bullet fragments that partially obscured vessel assessment.

When analyzing the flowchart consisting of indications and imaging in penetrating trauma to the extremities, CTA may be over utilized at our institute, since more than half, 57.0%, were negative for vascular injury, and slightly more often in GSW 60.4% compared to SW 53.7%. When assessing the patients’ medical records, we found that most of the studies had normal vascular exams, with hard signs of vessel injury recorded in only 13.4%, all of which underwent acute surgery. ABI-index in the negative CTAs were documented in 30.6%, and were all normal, except one with increased ABI-index. None of the negative CTAs required acute surgery, all were managed through conservative treatment, and no complications occurred within the 30-day study period.

In instances where a physical examination alone does not conclusively diagnose or exclude an injury, CTA is the preferred imaging technique for assessing potential vascular damage. It is fast, readily available, with high sensitivity and specificity [2, 3, 8, 12, 16]. Numerous prior studies have thoroughly investigated the sensitivity and specificity of CTA in the context of penetrating trauma. These investigations have consistently demonstrated a high sensitivity, 90–95.1%, and specificity 98.7–100%, for the identification of vascular injuries in extremities [17]. In our material the high sensitivity for vascular injury was equal to previous studies, 85.69–100%, however the specificity was lower 61.46–81.88%. We attribute the latter finding in our study to the broader indications for CTA that were implemented at our institute during the study period. Without relevant findings on clinical examination there is a risk of over diagnosing vascular injury on CTA that does not warrant surgical intervention.

In reviewing previous studies, some contradict the use of CTA as a screening method for traumatic vessel injuries to the extremities [9, 11, 12, 18]. Our findings do not dispute this conclusion. CTA, when performed without relevant clinical indication, presents with a risk for unnecessary radiation to the patient, over diagnosis, and identification of incidental lesions with unnecessary follow up and cost.

However, in the setting of potential occult vessel and the impact on wound and fracture healing, CTA has been shown to prove favorable in predicting poor patient outcomes [8]. In addition, CTA performed in the initial WBCT has the additional benefit of shorter decision times for surgery, observation times, hospital stays, and alleviating follow up [13]. Furthermore, CTA can provide critical additional details about osseous and soft-tissue injuries, including trajectory and relation to an injured vessel [4]. Our findings are in accordance with this. From a radiation standpoint, the information provided with CTA outperforms NECT, CECT and XR in penetrating traumatic injuries to the extremity. However, unnecessary imaging should be avoided if clinical examination is sufficient for proper clinical management. In our study we found that GSW had a higher probability for acute surgery, and complications compared to SW. However, more than half of CTAs performed were negative. Considering this, it is important to stratify patients into exam-based indications for CTA, and in such to weigh the risks of radiation with the benefits of ruling out clinically significant vascular injury, occult bleedings and when there is need to visualize osseous and soft-tissue injuries and localization of bullet fragments.

### Limitations

Limitations of this study include the retrospective design and data from a single institution.

In our study, DSA or surgical intervention with entry of vascular repair in the patient medical record, was the reference standard of CTAs positive for vessel injury. Considering our cohort, all positive CTAs had vessel injuries verified intra-operatively. However, negative CTAs with vessel injuries that could have been missed, and which did not develop complications in need of surgery or additional imaging could have falsely been classified as a true negative. In our study, none of the true negative CTAs developed any complications. Regarding the clinical outcome, false-positive CTAs are a risk of unnecessary invasive procedures and follow up. In our study, 19 cases were false positive, all treated conservatively, of which 2 developed compartment syndrome and required fasciotomy.

Finally, data was not available for some patients as some avoided follow-up, were incarcerated, or presented in the emergency room without a social security number which prevented further pursuit in patient medical records.

## Conclusions

In a Scandinavian Level-1 Trauma Center, about every fourth to fifth admission for penetrating trauma involved penetrating extremity injuries. GSWs had a significantly higher probability for acute surgery, and higher rate of 30-day complications. The rate of arterial injury was around one third of all injuries. CTA is effective and sensitive in detecting clinically relevant vascular injuries. Half of CTAs were negative. Stratifying patients into exam-based indication for CTA is important to reduce unnecessary imaging. Imaging should be reserved for when additional information, such as suspicion of occult bleeding, proximity to major arteries, bullet fragments, or assessment of osseus and soft tissue injuries, is needed for added clinical or surgical value.

## Data Availability

Data is available upon reasonable request. Further data can be obtained by email request to the corresponding author.

## Abbreviations

CECT: Contrast enhanced CT
CM: Contrast media
CT: Computed tomography
CTA: CT angiography
DSA: Digital subtraction angiography
GSW: Gunshot wounds
HU: Hounsfield unit
ISS: Injury severity score
IV: Intra venous
NECT: Non-enhanced CT
NISS: New injury severity score
OR: Operating room
OSW: Other stab wounds
PACS: Picture archiving and communication system
ROI: Region of interest
SW: Stab wounds
TRD: Trauma registry database
WBCT: Contrast enhanced whole-body CT
XR: X-ray imaging
